# The prognostic role of the AST/ALT ratio in hepatocellular carcinoma patients receiving thermal ablation combined with simultaneous TACE

**DOI:** 10.1186/s12876-023-02719-1

**Published:** 2023-03-21

**Authors:** Feihang Wang, Shanshan Gao, Mengfei Wu, Danyang Zhao, Huiyi Sun, Sothea Yav, Yi Chen, Zihan Zhang, Minjie Yang, Yi Dong, Jianhua Wang, Xiaolin Wang, Zhiping Yan, Lingxiao Liu

**Affiliations:** 1 Department of Interventional Radiology, Zhongshan Hospital, Fudan University, Shanghai Institute of Medical Imaging, No.180 Fenglin Road, Xuhui District, Shanghai, 200032 China; 2 National Clinical Research Center for Interventional Medicine, Zhongshan Hospital, Fudan University, Shanghai, China; 3 Department of Radiology, Zhongshan Hospital, Fudan University, Shanghai, 200032 China; 4 Department of CT&MRI, Third Hospital of Hebei Medical University, , Shijiazhuang, 050051 Hebei Province China; 5 Department of Ultrasound, Zhongshan Hospital, Fudan University, Shanghai, China

**Keywords:** TACE, Thermal ablation, Hepatocellular carcinoma, Enzymes

## Abstract

**Background:**

To evaluate the prognostic value of the pre-treatment aspartate transaminase (AST)/alanine transaminase (ALT) ratio in hepatocellular carcinoma (HCC) patients receiving radiofrequency ablation (RFA)/microwave ablation (MWA) combined with simultaneous TACE.

**Methods:**

The data for 117 patients were retrospectively analyzed in this study. The endpoint of prognosis was overall survival (OS). The Youden index was used to choose the optimal cut-off value of the pre-treatment AST/ALT ratio for OS prediction. Univariate and multivariate analyses were used to identify independent risk factors, then integrated to establish the nomogram.

**Results:**

The AST/ALT ratio cut-off value for OS prediction was 0.89, and patients with a higher AST/ALT ratio had poorer OS. The median OS for the high-value AST/ALT group was not reached, while the median OS for the low-value AST/ALT group was 48.5 months (*P* = 0.0047). The univariate and multivariate analysis showed that AST/ALT ratio, AFP, and tumor numbers were independent prognostic indicators for OS. The integrated nomogram showed higher predictive accuracy for OS (C-index 0.674, 95%CI: 0.600–0.748).

**Conclusions:**

The preoperative AST/ALT ratio could be a prognostic indicator for HCC patients receiving thermal ablation combined with simultaneous TACE.

## Background

Hepatocellular carcinoma (HCC) is still one of the most prevalent malignant tumors worldwide. The prognosis of HCC is poor, and the mortality rate of HCC continues to increase at an annual rate of approximately 2%-3% [1]. The main HCC treatments include surgical resection, liver transplantation, ablation, transarterial chemoembolization (TACE), and systemic treatment [2]. Although surgeries, including surgical resection and liver transplantation, are curative treatments, less than 20% of patients are suitable candidates [3]. Locoregional therapies include TACE, radiofrequency ablation (RFA), percutaneous ethanol injection (PEI), microwave ablation (MWA), high-intensity focused ultrasound (HIFU), and others. TACE is one of the recommended therapies for intermediate-stage HCC according to the Barcelona clinic liver cancer (BCLC) staging classification [2]. However, the tumor necrosis rate is low, and the intrahepatic recurrence rate is high applying TACE alone [4]. Percutaneous thermal ablation, including RFA and MWA, is considered the optimum therapy choice for focal unresectable early-stage HCC. It can cause coagulation necrosis of tumors and allow a long-term survival rate comparable to surgical resection [5]. Ablation is hard to achieve complete tumor necrosis when it comes to large lesions or tumors adjacent to a large vessel. The combination of TACE with MWA or RFA has advantages compared to monotherapy and can complement the disadvantages of each other [3, 6, 7]. However, the number of prognostic and predictive models in HCC patients undergoing thermal ablation combined with simultaneous TACE is limited.

The serum transaminases, such as aspartate transaminase (AST) and alanine transaminase (ALT), are routine laboratory markers for liver function. In 2015, Bezan showed that a high preoperative AST/ALT ratio (termed the De Ritis ratio) was a prognostic factor for nonmetastatic renal cell carcinoma [8]. Since then, some studies have examined the prognostic value of the AST/ALT ratio in various cancers, such as prostate cancer, oropharyngeal cancer, colorectal carcinoma, and HCC [9–11]. Zhang et al. [12] showed that high AST/ALT ratio was significantly correlated with poorer OS in primary HCC patients. In addition, Liu et al. [13] depicted that high aspartate aminotransferase-to-alanine aminotransferase ratio (AAR) implied poor OS in HCC patients undergoing TACE. However, to the best of our knowledge, there are no studies on AST/ALT in predicting the prognosis of HCC patients who received thermal ablation combined with simultaneous TACE.

This study aimed to examine the value of pre-treatment AST/ALT in the prognosis of HCC patients who received thermal ablation combined with simultaneous TACE and establish a prognosis model by developing a nomogram incorporating AST/ALT ratio into BCLC staging classification.

## Methods

### Patients

The data for 117 patients who received percutaneous thermal ablation combined with simultaneous TACE procedure in Zhongshan Hospital, affiliated with Fudan University, from November 2010 to January 2017 were retrospectively collected. The inclusion criteria were as follows: (1) diagnosis of hepatocellular carcinoma based on pathological diagnosis or imaging diagnosis; (2) no major vascular invasion, portal vein thrombosis, or extrahepatic metastasis; (3) Patients with primary or recurrent HCC who were not candidates or refused surgical resection; (4) Child–Pugh Class A or B; (5) platelet count > 50 × 10^9/L, prothrombin time ≤ 18 s. The exclusion criteria for the study were as follows: (1) incomplete medical records; (2) lost to follow-up; (3) major vascular invasion,portal vein thrombosis os extrahepatic metastasis; (4) Child–Pugh Class C.

### Pre-treatment evaluation

Enhanced computed tomography (CT) or magnetic resonance imaging (MRI) of the abdomen was performed two weeks before the operation to determine the location, number, size, and relationship between the tumor and adjacent tissues. Ultrasound (US) was performed to judge the visibility of tumors. Blood routine, coagulation function, liver and kidney function, tumor markers, and other related laboratory tests were done within one week before the operation.

### Thermal ablation combined with simultaneous TACE procedure

The patient was placed in the supine position during the operation with conscious sedation and/or local anesthesia. Based on the pre-treatment radiological image and the angiography result, a 14G water‑cooled MWA antenna (Nanjing Vison‑China Medical Devices R and D Center, China) or 17G RFA electrode needle (MedSphere Shanghai, China) was inserted into the tumor through percutaneous puncture with US guidance. The microwave power was set between 80-100W. The radiofrequency adopted the automatic impedance mode with the power set at 120W. The maximum temperature was set at 110 °C. Single-point ablation or multi-point overlapping ablation was used according to the size of the lesions and the ablation zone needed to cover the lesion completely. Suppose the lesion was more than 0.5 cm away from difficult locations (large blood vessels, bile ducts, and hollow organs). In that case, the ablation area should exceed the edge of the tumor by 0.5–1.0 cm to achieve a safe margin. If the tumor was close to the difficult locations, it is not mandatory to have the ablation area exceed the 0.5–1.0 cm tumor region. After the ablation was completed, the power was lowered to 50W, and needle tract ablation was carried out to prevent needle tract bleeding and tumor implantation metastasis.

The femoral artery or radial artery was punctured with a 5F-sheath using the modified Seldinger method. A 4-5F RH catheter was used to select the celiac trunk and/or superior mesenteric angiography for angiography to observe the results of ablation, residual lesions, bleeding, and arteriovenous fistula. Microcatheter super-selective catheterizations were performed in the target artery. The emulsion, which was made with platinum-based chemotherapy (100-150 mg) (Jiangsu Hengrui Medicine, Lianyungang, China) + epirubicin (30-50 mg) (Farmorubicin; Pfizer, Wuxi, China) + lipiodol (5–10 ml) (Guerbet, Roissy, France) + Embosphere 500 μm or less (Merit Medical Systems, Inc., South Jordan, UT, USA), was injected into the target artery with digital subtraction angiography (DSA) fluoroscopy. Super-selective gelatin sponge particles were given for embolization if arterial-portal venous fistula or arterial-hepatic venous fistula occurred. A microcatheter was used to select the target artery and embolize with coils for arterial hemorrhage.

### Follow-up

Patients received abdominal enhanced CT or MRI and laboratory tests (blood routine, liver and kidney function, tumor markers, etc.) at 1-, 3-, 6-, 9-, and 12-month after the operation. Patients were followed up every six months thereafter if no active intrahepatic lesions or extrahepatic metastasis were found in the imaging examination within one year. If there were active intrahepatic lesions or extrahepatic metastasis, TACE, ablation, systemic therapy, or other treatments tailored to the patient’s situation were chosen. The end of follow-up is the follow-up deadline of December 31, 2020, or the patient’s date of death. The endpoint of the analysis was overall survival (OS), defined as the time from receiving thermal ablation combined with simultaneous TACE for the first time to the end of follow-up.

### Statistical analysis

SPSS statistical software (version 24.0) was used for data analysis in this study. Continuous variables were expressed as mean ± standard deviation, and categorical variables were expressed as percentages. The Youden Index was used to calculate the cut-off value of the pre-treatment AST/ALT ratio. The Kaplan–Meier method was used to draw a survival curve to calculate the cumulative survival rate. Log-rank test was used for univariate analysis, and the Cox regression model was used for multivariate analysis. Factors in univariate analysis with *P* < 0.05 were included in multivariate analysis. *P* < 0.05 was considered statistically significant in both the univariate and multivariate analysis. The package of rms in R (Version 4.1.0) was used to draw a nomogram of prognosis according to the results of multivariable analysis, and further evaluated the performance of the nomogram by the C-index.

## Results

### Analysis at baseline

Clinicopathological characteristics of patients were presented in Table 1. Generally, among the 117 included patients, 96 (82.1%) were male, and 21 (17.9%) were female. The median age was 60 ± 10 years. Most patients received MWA combined with simultaneous TACE (*n* = 79, 67.5%) and the other patients underwent RFA combined with simultaneous TACE (*n* = 38, 32.5%).

**Table 1:** Baseline characteristics of the research patients (*n* = 117)

General information	Data
Age(y)	60 ± 10
Sex [n (%)]	
Male	96 (82.1%)
Female	21 (17.9%)
Operation mode [n (%)]	
RFA + TACE	38 (32.5%)
MWA + TACE	79 (67.5%)
Hepatectomy before treatment [n (%)]	
Yes	25 (21.4%)
No	92 (78.6%)
Hepatitis [n (%)]	
HBV	102 (87.2%)
HCV	4 (3.4%)
Cirrhosis [n (%)]	
yes	80 (68.4%)
no	37 (31.6%)
ALB(g/l)	40.0 ± 5.0
AFP (ng/ml)	497.5 ± 1776.8
AST/ALT ratio	1.3 ± 1.3
Tumor numbers	
1	68 (58.1%)
2	18 (15.4%)
≥ 3	31 (26.5%)
Tumor maximum diameter(mm)	27.7 ± 12.9
BCLC stages	
A	86 (73.5%)
B	31 (26.5%)
Child–Pugh grades	
A	113 (96.6%)
B	4(3.4%)

*RFA* radiofrequency ablation, *TACE* transarterial chemoembolization, *MWA* microwave ablation, *HBV* hepatitis B virus, *HCV* hepatitis C virus, *ALB* albumin, *AFP* alpha fetoprotein, *AST* aspartate transaminase, *ALT* alanine transaminase, *BCLC* Barcelona Clinic Liver Cancer

### Relationships between AST/ALT ratio and other characteristics

The median AST/ALT ratio was 1.3. Stratified by the Youden index, the optimal cut-off value for OS prediction was 0.89. All patients included in the study were divided into two groups according to AST/ALT ratio: a high-value group (AST/ALT > 0.89, *n* = 85) and a low-value group (AST/ALT ≤ 0.89, *n* = 32). The low-value AST/ALT group was associated with a lower ALT count (*p* < 0.001) and lower AFP (*p* = 0.013). In addition, a statistically significant association was found in sex between two groups (*p* = 0.01) **(**Table 2).

**Table 2:** Correlation between AST/ALT ratio and clinicopathological variables of patients with hepatocellular carcinoma

Variables	AST/ALT ≤ 0.89 (*n* = 32)	AST/ALT > 0.89 (*n* = 85)	*p*
Sex, female/male	1/31	20/65	0.01^*^
Age, < 60/ ≥ 60	18/14	31/54	0.061
Operation mode, MWA + TACE/RFA + TACE	19/13	60/25	0.248
Hepatectomy before treatment, No/Yes	22/1	70/15	0.11
HBV, No/Yes	3/29	12/73	0.709
HCV, No/Yes	30/2	83/2	0.643
Cirrhosis, No/Yes	14/18	23/62	0.084
ALB, < 35/ ≥ 35	2/3	11/74	0.486
ALT, ≤ 40/ > 40	15/17	76/9	< 0.001^*^
AST, ≤ 35/ > 35	21/11	56/29	0.979
AFP, < 200/ ≥ 200	29/3	58/27	0.013^*^
Number, < 3/ ≥ 3	26/6	60/25	0.244
Tumor maximum diameter, < 30/ ≥ 30	23/9	51/34	0.235
Tumor maximum diameter, < 50/ ≥ 50	32/0	74/11	0.075
BCLC, A/B	27/5	59/26	0.102
Child–Pugh grade, A/B	32/0	81/4	0.498

*RFA* radiofrequency ablation, *TACE* transarterial chemoembolization, *MWA* microwave ablation, *HBV* hepatitis B virus, *HCV* hepatitis C virus, *ALB* albumin, *AFP* alpha fetoprotein, *AST* aspartate transaminase, *ALT* alanine transaminase, *BCLC* Barcelona Clinic Liver Cancer

^*^Data are statistically significant results

### Analysis of the primary endpoint: OS

**Fig. 1:**
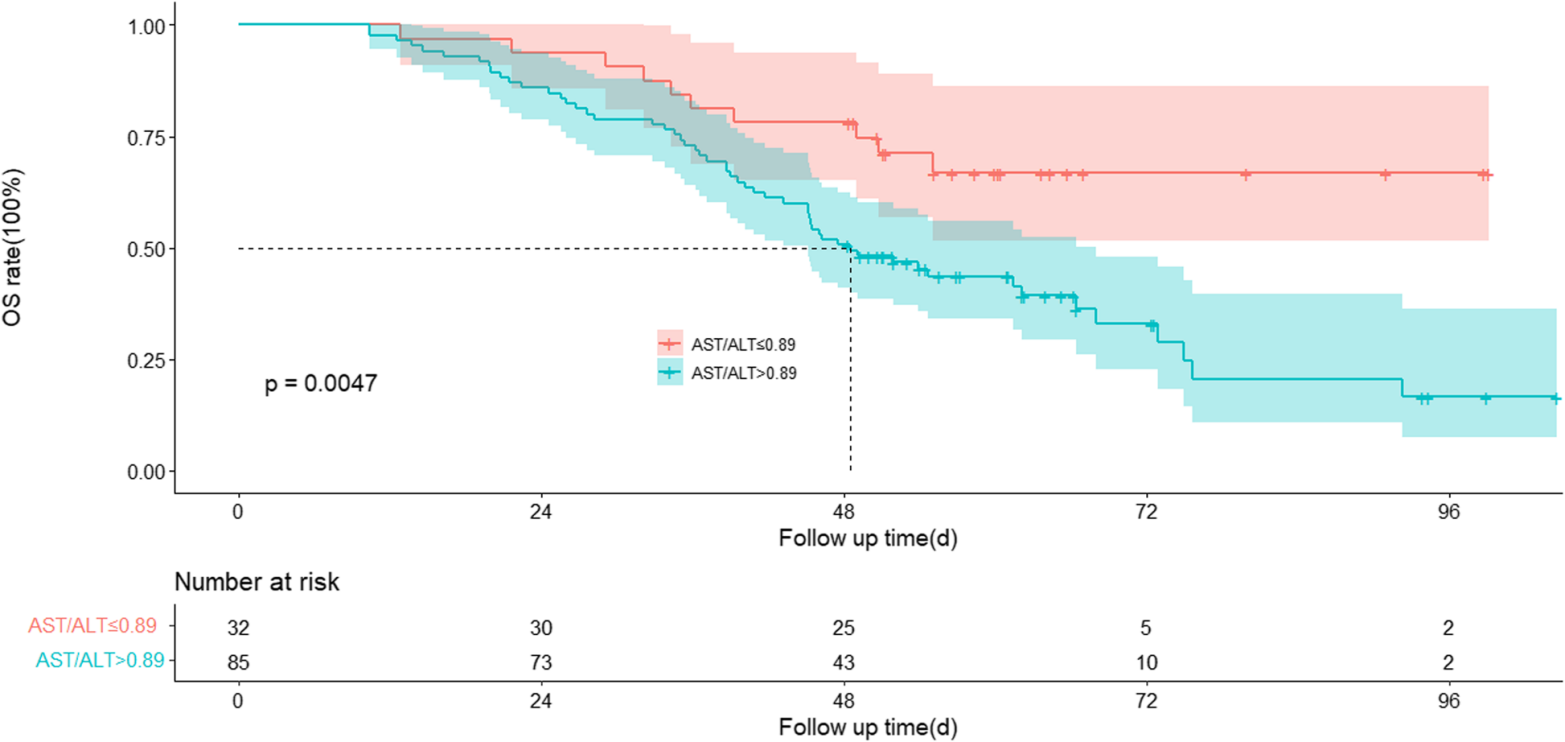
Kaplan–Meier survival curve for patients with hepatocellular carcinoma according to the AST/ALT ratio

The median follow-up time was 49.8 ± 20.6 months. By the end of follow-up, 65 patients had died. The median OS was 55.1 months (95% CI: 41.0–69.1 months). The 1-, 3-, 5-, and 7-year cumulative survival rates were 98.0%, 75.0%, 50.0%, and 32.0%, respectively. The median OS for the high-value AST/ALT group was not reached, while the median OS for the low-value AST/ALT group was 48.5 months (*P* = 0.0047) **(**Fig. 1). Furthermore, univariate analysis showed that AFP (*P* = 0.015), tumor numbers (*P* < 0.001), maximum tumor diameter (*P* = 0.01), and BCLC stage (*p* < 0.001) were statistically correlated with OS. In multivariate analysis, AFP (HR: 1.961; 95%CI: 1.140–3.374; *P* = 0.015), AST/ALT ratio (HR: 2.075; 95%CI: 1.039–4.145; *P* = 0.039) and tumor numbers (HR: 3.074; 95%CI: 1.832–5.160; *P* < 0.001) were identified as independent prognostic indicators for OS (Table 3).

**Table 3:** Univariate and multivariate analyses of clinicopathological characteristics of patients with hepatocellular carcinoma for OS

Parameter	Patient (*n* = 117)	OS		
		Univariate *P*-value	Multivariate *P*-value	HR (95%CI)
Age (y)				
< 60	49			
≥ 60	68	0.669		
Sex				
Male	96			
Female	21	0.13		
Operation mode				
RFA + TACE	38			
MWA + TACE	79	0.77		
Hepatectomy before treatment				
YES	25			
NO	92	0.237		
Cirrhosis				
YES	80			
NO	37	0.12		
ALB (g/l)				
< 35	13			
≥ 35	104	0.057		
AFP (ng/ml)				
< 200	87			
≥ 200	30	0.015^*^	0.015^*^	1.961(1.140–3.374)
AST/ALT ratio				
≤ 0.89	32			
> 0.89	85	0.005^*^	0.039^*^	2.075(1.039–4.145)
Tumor numbers				
< 3	86			
≥ 3	31	< 0.001^*^	< 0.001^*^	3.074(1.832–5.160)
Tumor maximum diameter(mm)				
< 50	106			
≥ 50	11	0.01^*^	0.22	
BCLC stage				
A	86			
B	31	< 0.001^*^	0.891	
Child–Pugh grades				
A	113			
B	4	0.156		

*OS* overall survival, *RFA* radiofrequency ablation, *TACE* transarterial chemoembolization, *MWA* microwave ablation, *ALB* albumin, *AFP* alpha fetoprotein, *AST* aspartate transaminase, *ALT* alanine transaminase, *BCLC* Barcelona Clinic Liver Cancer

^*^Data are statistically significant results

### Prognostic nomograms integrating AFP, tumor number, AST/ALT ratio and BCLC

**Fig. 2:**
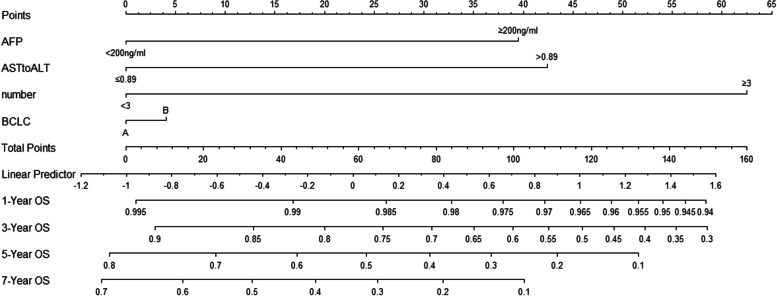
Nomogram predicts 1-,3-,5-and 7-year OS in hepatocellular carcinoma patients

A model was established by integrating AFP, tumor number, AST/ALT ratio, and BCLC **(**Fig. 2). The C-index for OS prediction of the formulated nomogram was 0.674 (95%CI: 0.600–0.748), which was higher than BCLC (0.607, 95%CI:0.548–0.666) and other indicators and combinations (Table 4).

**Table 4:** The identification ability and independent prognostic factors of the nomogram of patients with hepatocellular carcinoma: concordance indices in overall survival prediction

Variables	OS	
	C-index	95%CI
AST/ALT ratio	0.573	0.516–0.630
BCLC	0.607	0.548–0.666
AFP	0.564	0.505–0.623
number	0.62	0.563–0.677
BCLC + AFP + number	0.657	0.584–0.730
BCLC + AFP + number + AST/ALT ratio	0.674	0.600–0.748

*OS* overall survival, *AST* aspartate transaminase, *ALT* alanine transaminase, *AFP* alpha fetoprotein, *BCLC* Barcelona Clinic Liver Cancer

## Discussion

This study found that the AST/ALT ratio was an easily accessible and novel prognostic factor for HCC patients receiving thermal ablation combined with simultaneous TACE. A high AST/ALT ratio was significantly associated with poor outcomes expressed as OS. Furthermore, the nomogram integrating AST/ALT ratio, AFP, tumor number, and BCLC had improved predictive ability compared with the BCLC staging classification alone.

Since Fernando De Ritis first described AST/ALT ratio as an enzymatic test for viral hepatitis in 1957 [14], researchers have taken it as a potential prognostic biomarker in various diseases [8–13, 15, 16]. Also, Granito et al. [17] evaluated the prognostic significance of changes in liver parameters as predictors of treatment response to cTACE. They found that a postprocedure increase of transaminases (AST increase ≥ 46%, ALT increase ≥ 52%) compared with baseline values was shown to be a reliable predictor of response to cTACE. The results proved the transaminase value as prognostic indicators of HCC treatments. AST and ALT are important enzymes in the liver and are widely used to evaluate liver function. AST is mainly found in the mitochondria of liver cells, while ALT is presented primarily outside the mitochondria of liver cells. The serum AST and ALT can be impacted by many non-tumor-related factors, such as coronary heart disease, chronic hepatitis, and drugs. Therefore, the combination of AST and ALT as AST/ALT ratio is more stable and appropriate than they were used as single predictors [18]. Cancer cells have a higher rate of aerobic glycolysis to generate more energy to support their high proliferative status than normal cells [19]. AST plays a vital role in aerobic glycolysis due to its ability to relocate nicotinamide adenine dinucleotide hydrogen into mitochondria through malate-aspartate shuffling [20]. Thus, more AST is activated than ALT in high proliferative cancer tissues. Moreover, glutaminolysis and pyruvate production are catalyzed by ALT, which are also strengthened in tumor cells [21]. Hence, the AST/ALT ratio may reflect the metabolic status in cancers, possibly related to tumor growth and progression.

Therefore, we hypothesized that the AST/ALT ratio maybe a potential prognostic indicator for HCC patients receiving thermal ablation combined with simultaneous TACE in our study. According to the optimal cut-off value of AST/ALT ratio, we stratified patients into two groups, and found the subgroup with lower value of AST/ALT ratio (≤ 0.89) was related to the lower ALT count and lower AFP. In addition, High AST/ALT ratio was associated with poor OS, consistent with the literature. Wu et al. [18] analyzed 18 studies and concluded that an elevated serum AST/ALT ratio before treatment was significantly associated with poor clinical outcomes of OS in patients with solid tumors. Another study also depicted that a high AST/ALT ratio implied poor OS in HCC patients accepting TACE [13].

AFP is the most frequently used biomarker for HCC [22]. It has been proven that constantly increased serum AFP level was correlated with aggressive histological morphology of HCC, such as vascular invasion, satellitosis and poorly differentiated [23–25]. A previous study had demonstrated that elevated AFP meant dismal prognosis [26]. However, excluding the liver transplantion, an AFP threshold has not been used as a guide for HCC treatment. Till now, the decision -making process mainly relies on imaging features and clinical characteristics without consideration of markers of tumor aggressiveness. This approach may hinder the optimal management of HCC patients, since there are increasing evidences suggesting that cancer biology is a helpful predictor of treatment outcomes [27]. Potentially, AFP represents an easily accessable biomarker to modify the decision-making process currently adopted for the management of HCC. Herein, we integrated AFP, number, BCLC, and AST/ALT ratio and improved the predictive accuracy of BCLC system in terms of C-index.

There were some shortcomings in our study. First, this was a single-center retrospective study with small sample size. Due to the characteristics of the retrospective study, some biases were hard to be avoided. Second, this study did not exclude patients who have previously received locoregional treatment, which might impact the result. Third, some patients received additional treatments after first-time thermal ablation combined with simultaneous TACE, which might have a confounding effect on the tumor response and outcome.

Despite the limitations mentioned above, our study still had clinical value. First, it was the first time to show the predictive value of AST/ALT ratio in HCC patients receiving thermal ablation combined with simultaneous TACE. Second, AST/ALT ratio was a conventional blood parameter, and its convenience, easy accessibility and inexpressiveness revealed its excellent performance in risk stratification for HCC patients. In addition, AST/ALT ratio could become an effective tool for future clinical studies and patient management.

## Conclusions

This study investigated the prognostic role of the AST/ALT ratio in the outcome of HCC patients undergoing thermal ablation combined with simultaneous TACE. The AST/ALT ratio was an independent prognostic factor for HCC patients receiving thermal ablation combined with simultaneous TACE, and a high preoperative AST/ALT ratio indicated poor OS. Besides, the constructed nomogram including AFP, number, BCLC, and AST/ALT ratio was confirmed as a well-performed prognostic model for OS. This finding may help improve the management of HCC patients who accept thermal ablation combined with simultaneous TACE through early identification of patients with potential poor outcomes.

## Data Availability

The datasets used and/or analyzed during the current study are available from the corresponding author on reasonable request.

## Abbreviations

AST: Aspartate transaminase
ALT: Alanine transaminase
HCC: Hepatocellular carcinoma
RFA: Radiofrequency ablation
MWA: Microwave ablation
OS: Overall survival
PFS: Progression-free survival
AFP: Alpha fetoprotein
TACE: Transarterial chemoembolization
PEI: Percutaneous ethanol injection
HIFU: High-intensity focused ultrasound
BCLC: Barcelona Clinic Liver Cancer
mRECIST: The modified response evaluation criteria in solid tumors
HBV: Hepatitis B virus
HCV: Hepatitis C virus
ALB: Albumin

## References

[1] Wang W, Wei C (2020). Advances in the early diagnosis of hepatocellular carcinoma. Genes Dis..

[2] Reig M, Forner A, Rimola J (2022). BCLC strategy for prognosis prediction and treatment recommendation: the 2022 update. J Hepatol.

[3] Xu Z, Xie H, Zhou L, Chen X, Zheng S (2019). The combination strategy of transarterial chemoembolization and radiofrequency ablation or microwave ablation against hepatocellular carcinoma. Anal Cell Pathol (Amst)..

[4] Li W, Ni CF (2019). Current status of the combination therapy of transarterial chemoembolization and local ablation for hepatocellular carcinoma. Abdom Radiol (NY)..

[5] Lencioni R, Crocetti L (2012). Local-regional treatment of hepatocellular carcinoma. Radiology.

[6] Gui CH, Baey S, D’cruz RT, Shelat VG (2020). Trans-arterial chemoembolization + radiofrequency ablation versus surgical resection in hepatocellular carcinoma - A meta-analysis. Eur J Surg Oncol..

[7] Wu M, Gao S, Song H (2019). Percutaneous thermal ablation combined with simultaneous transarterial chemoembolization for hepatocellular carcinoma </=5 cm. J Cancer Res Ther.

[8] Bezan A, Mrsic E, Krieger D (2015). The preoperative AST/ALT (De Ritis) ratio represents a poor prognostic factor in a cohort of patients with nonmetastatic renal cell carcinoma. J Urol.

[9] Zhou J, He Z, Ma S, Liu R (2020). AST/ALT ratio as a significant predictor of the incidence risk of prostate cancer. Cancer Med..

[10] Knittelfelder O, Delago D, Jakse G (2020). The AST/ALT (De Ritis) ratio predicts survival in patients with oral and oropharyngeal cancer. Diagnostics (Basel)..

[11] Scheipner L, Smolle MA, Barth D (2021). The AST/ALT ratio is an independent prognostic marker for disease-free survival in stage II and III colorectal carcinoma. Anticancer Res.

[12] Zhang LX, Lv Y, Xu AM, Wang HZ (2019). The prognostic significance of serum gamma-glutamyltransferase levels and AST/ALT in primary hepatic carcinoma. BMC Cancer.

[13] Liu C, Jia BS, Zou BW (2017). Neutrophil-to-lymphocyte and aspartate-to-alanine aminotransferase ratios predict hepatocellular carcinoma prognosis after transarterial embolization. Medicine (Baltimore).

[14] Ritis de F, Coltorti M, Giusti G (1957). An enzymic test for the diagnosis of viral hepatitis; the transaminase serum activities. Clin Chim Acta..

[15] Shijo H, Okazaki M (1991). Prediction of portal vein invasion by hepatocellular carcinoma: a correlations between portal vein tumor thrombus and biochemical tests. Jpn J Clin Oncol..

[16] Shen J, Dai J, Zhang Y (2021). Baseline HBV-DNA load plus AST/ALT ratio predicts prognosis of HBV-related hepatocellular carcinoma after hepatectomy: a multicentre study. J Viral Hepat.

[17] Granito A, Facciorusso A, Sacco R (2021). TRANS-TACE: prognostic role of the transient hypertransaminasemia after conventional chemoembolization for hepatocellular carcinoma. J Pers Med..

[18] Wu J, Chen L, Wang Y, Tan W, Huang Z (2019). Prognostic value of aspartate transaminase to alanine transaminase (De Ritis) ratio in solid tumors: a pooled analysis of 9,400 patients. Onco Targets Ther..

[19] Dang CV (2012). Links between metabolism and cancer. Genes Dev..

[20] Sookoian S, Pirola CJ (2015). Liver enzymes, metabolomics and genome-wide association studies: from systems biology to the personalized medicine. World J Gastroenterol..

[21] Friday E, Oliver R, Welbourne T, Turturro F (2011). Glutaminolysis and glycolysis regulation by troglitazone in breast cancer cells: relationship to mitochondrial membrane potential. J Cell Physiol..

[22] Hu X, Chen R, Wei Q, Xu X (2022). The Landscape Of Alpha Fetoprotein In Hepatocellular Carcinoma: Where Are We?. Int J Biol Sci..

[23] Forner A, Reig M, Bruix J (2018). Hepatocellular carcinoma. Lancet..

[24] Galle PR, Foerster F, Kudo M (2019). Biology and significance of alpha-fetoprotein in hepatocellular carcinoma. Liver Int.

[25] Peng SY, Chen WJ, Lai PL, Jeng YM, Sheu JC, Hsu HC (2004). High alpha-fetoprotein level correlates with high stage, early recurrence and poor prognosis of hepatocellular carcinoma: significance of hepatitis virus infection, age, p53 and beta-catenin mutations. Int J Cancer..

[26] Cabibbo G, Maida M, Genco C (2012). Natural history of untreatable hepatocellular carcinoma: a retrospective cohort study. World J Hepatol.

[27] Trevisani F, Garuti F, Neri A,  (2019). Alpha-fetoprotein for diagnosis, prognosis, and transplant selection. Semin Liver Dis.

